# Combining macula clinical signs and patient characteristics for age-related macular degeneration diagnosis: a machine learning approach

**DOI:** 10.1186/1471-2415-15-10

**Published:** 2015-01-27

**Authors:** Paolo Fraccaro, Massimo Nicolo, Monica Bonetto, Mauro Giacomini, Peter Weller, Carlo Enrico Traverso, Mattia Prosperi, Dympna OSullivan

**Affiliations:** Centre for Health Informatics, City University London, London, UK; Centre for Health Informatics, University of Manchester, Manchester, UK; DIBRIS, University of Genoa, Genoa, Italy; Di.N.O.G.Mi, University of Genoa, L.go P. Daneo 3, Genoa, 16132 Italy; CEBR, University of Genoa, Genoa, Italy; NIHR Primary Care Patient Safety Translational Research Centre, University of Manchester, Manchester, UK; Health eResearch Centre, University of Manchester, Manchester, UK

**Keywords:** Age related macular degeneration, Machine learning, Automated diagnosis, Statistical learning, macula disease

## Abstract

**Background:**

To investigate machine learning methods, ranging from simpler interpretable techniques to complex (non-linear) “black-box” approaches, for automated diagnosis of Age-related Macular Degeneration (AMD).

**Methods:**

Data from healthy subjects and patients diagnosed with AMD or other retinal diseases were collected during routine visits via an Electronic Health Record (EHR) system. Patients’ attributes included demographics and, for each eye, presence/absence of major AMD-related clinical signs (soft drusen, retinal pigment epitelium, defects/pigment mottling, depigmentation area, subretinal haemorrhage, subretinal fluid, macula thickness, macular scar, subretinal fibrosis). Interpretable techniques known as white box methods including logistic regression and decision trees as well as less interpreitable techniques known as black box methods, such as support vector machines (SVM), random forests and AdaBoost, were used to develop models (trained and validated on unseen data) to diagnose AMD. The gold standard was confirmed diagnosis of AMD by physicians. Sensitivity, specificity and area under the receiver operating characteristic (AUC) were used to assess performance.

**Results:**

Study population included 487 patients (912 eyes). In terms of AUC, random forests, logistic regression and adaboost showed a mean performance of (0.92), followed by SVM and decision trees (0.90). All machine learning models identified soft drusen and age as the most discriminating variables in clinicians’ decision pathways to diagnose AMD.

**Conclusions:**

Both black-box and white box methods performed well in identifying diagnoses of AMD and their decision pathways. Machine learning models developed through the proposed approach, relying on clinical signs identified by retinal specialists, could be embedded into EHR to provide physicians with real time (interpretable) support.

**Electronic supplementary material:**

The online version of this article (doi:10.1186/1471-2415-15-10) contains supplementary material, which is available to authorized users.

## Background

Age-related macular degeneration (AMD) is the leading cause of severe reduction in central visual acuity in adults aged 50 years and older in developed countries [1]. As the prevalence of AMD is steadily increasing due to increasing life expectancy [2], early diagnosis and treatment becomes essential in slowing down progression of AMD and subsequent vision loss [3]. Multimodal high-resolution imaging has had a substantial impact on diagnosis and treatment of macular diseases [4]. Different imaging modalities can be used for AMD diagnosis [5]. In particular, optical coherence tomography (OCT) associated with color fundus image acquisition technology is a non-contact, non-invasive, high resolution technique which produces real-time images used to derive several features of the macula [6]. Such characteristics may allow OCT to become an effective screening instrument, employable in non-specialized environments (such as pharmacies) and by non-specialized personnel to perform automatic diagnosis of AMD without the intervention of a medical retinal specialist. However, to allow diagnosis by non-specialized personnel, OCT technology could be coupled with other clinical decision support functionalities [7] based on patient data which could enhance the potential of image analysis data. Currently, the majority of commercially available OCT technologies incorporate basic algorithms to automatically identify the presence of risk factors in macula images and diagnose AMD [8–10]. A recent review [5] showed how the majority of these algorithms mainly focus on automatic segmentation of soft drusen, previously identified as one of the most important signs for the diagnosis of AMD [11]. But relying on just one sign to diagnose AMD can be suboptimal since AMD is a complex pathology which involves different stages of progression and requires consideration of several clinical aspects [12]. Therefore image analysis should be used in conjunction with other clinical biomarkers to enhance diagnosis [5].

Machine learning techniques [13] have been applied successfully to identify, extract and analyze features in macula digital imaging [14–18]. In spite of potential higher accuracy in predicting disease diagnoses (as compared, for instance, to simple scoring rules on a small set of variables), many machine learning methods are usually regarded as non-transparent to the end user, and labeled as “black-boxes”. Methods that do not allow the clinician to identify a clear decision pathway for the diagnosis are often regarded with skepticism in the clinical community [14].

This paper describes the application of a variety of more and less interpretable machine learning algorithms with the aims of: 1) reproducing physicians’ diagnoses of AMD from patient data (demographics and clinical signs identified by retinal specialists through examination of medical images) and evaluating model performances on unseen data; 2) determining which are the diagnostic criteria followed by the physician (who may follow different routes to make a diagnosis); 3) identifying deviations or new rules that may emerge from the expected. Data were collected cross-sectionally from routine patient visits and stored in an Electronic Health Record, specifically designed for macular diseases management. The work reported in this paper is preliminary research into the potential of machine learning techniques to be used for AMD diagnostic support; from the perspective of using longitudinal data (i.e. symptoms/markers before a diagnosis is made), these decision support algorithms could be embedded in Electronic Health Records to support physicians during everyday clinical practice, and could be further refined by incorporating features from digital image processing leading to a fully-automated diagnostic tool suitable for non-specialized environments.

## Methods

### Ethics statement

The web Electronic Health Record system [19], data collection methods [20] and related observational studies were approved by an ethics committee (San Martino Hospital, Genoa, Italy) and patients signed informed consent for data storage and usage for clinical/research purposes.

### Study population and case selection

Data on study participants, healthy subjects (those with normal macula) as well as patients with macular diseases (both referred to as “patients” in the text), were collected from March 2013 to January 2014 during routine clinical practice at the Medical Retina Center of the University Eye Clinic of Genoa (Italy).

The attributes used in the analysis included patient’s age and gender, and for patient’s left/right eye’s:

- Primary diagnosis (AMD or other macular diseases);
- Relevant clinical signs [12] identified by clinicians during the visit as binary variables (positive if identified):
  - o Soft drusen;
  - o Retinal Pigment Epitelium (RPE) defects/pigment mottling;
  - o Depigmentation area(s);
  - o Subretinal haemorrhage;
  - o Subretinal fluid;
  - o Macula thickness;
  - o Macular scar;
  - o Subretinal fibrosis;

The macula of all eyes included in the study was evaluated by two different ophthalmologist (10 and 2 years of experience) using a spectral domain OCT machine (Topcon 3D OCT-2000, Topcon Medical Systems, Inc., Oakland, NJ, USA). Data records were stored per single eye.

The study population included a total of 487patients (912 eyes, with information on patients’ two eyes not always available).

Primary diagnosis of AMD is the study outcome (dependent variable). Accordingly, each eye observation diagnosed as AMD was assigned to one class, while eyes diagnosed with other macular diseases were assigned to another class. The covariate set (input variables) included all the other attributes listed above and referred to the same eye.

There is evidence in support of the hypothesis of disease correlation between different eyes of the same patient [21]. A preliminary screening on our data confirmed this hypothesis. However, information on the fellow eye may not be available when diagnoses are performed during a visit (for example, it may be the first encounter). Therefore, we performed the analysis in this paper without taking into account the information about the presence of AMD in the fellow eye.

### Machine learning techniques

The purpose of this section is not to provide a detailed explanation of machine learning methods, which is left to referenced works, but to give some introduction about the techniques which readers may be less familiar with [22]. All statistical analyses and graphs were done using the R software (http://www.r-project.org).

*Logistic regression* is used for predicting the outcome of a categorical dependent variable (i.e., a class label) based on one or more predictor variables. This “white-box” technique is widely used in automatic medical diagnosis [22, 23]. An embedded procedure within logistic regression, called the LogitBoost [24] (as implemented in *RWeka* R library [25]), was included to select the most relevant variables. No variable interactions were explored. For comparison purposes, a simple model based on a unique variable was employed, named “one-rule”, selecting the most discriminative variable, based on a univariable logistic regression fit.

*Support Vector Machines*[26] are classifiers that divide data instances of different categories with a linear boundary supported by a very clear gap (called maximum margin). They can be optimised via different internal algorithms, therefore a parameter search is often recommended. Support vector machines can efficiently perform a non-linear classification using a so-called “kernel trick” which maps their inputs into feature spaces of higher dimensions. This solution however is more difficult to interpret. In this study we adopted a linear kernel and the *nu-classification*, optimizing the parameter *nu* in the value range [0.02, 0.4], with a step size of 0.01 for values below 0.1, and a step of 0.05 for values above 0.1, using *e1071* library [27] in the R software.

*Decision trees* are non-linear graphical models that take the form of a flow chart. They are a “white-box” method because they produce multiple decision pathways in a tree form that can be easily interpretable [28]. Decision trees consist of nodes which represent input variables, and edges branching from the nodes dependent on possible values of those input variables. Each terminal node (leaf) represents the value of the target variable given the values of the input variables after following the path from the root to the leaf. A decision tree is usually *grown* by starting from the whole population, looking at the most discriminative variable to predict a desired outcome (which becomes a node), and splitting the data based on a cut-off value of this variable (inducing an edge). In our analysis, we adopted the *party* package of decision tree learning [29] within the R software.

A single decision tree often does not yield satisfactory prediction performance. To improve performance, multiple different trees can be aggregated, and this takes the general name of a *tree ensemble*. A weel-recognised tree ensemble method is the *random forest*[30], which infers different decision trees via resampling and randomization, producing an average prediction from all trees. We used the *randomForest* package of R [31]. The combination of several trees makes the method more powerful, but also more difficult to interpret than a single decision tree. Another ensemble method is the *AdaBoost*[32], which fits several “weak” learners, such as decision trees with only a small number of pathways, and weights them based on performance on data subsets. We adopted the *RWeka* AdaBoost version of R [25].

We performed the complete cases analysis with all methods and used three different approaches for imputation of missing values: i) addition of a categorical variable encoding the presence of a missing value; ii) substitution with the overall population mode for binary attributes and mean for numeric ones; iii) non-linear imputation based on random forests [33].

Models’ performance was analysed by means of sensitivity (true positive rate), specificity (true negative rate), and using the area under the receiver operating characteristic (AUC), which is a combined indicator of sensitivity and specificity, equal to the probability that a classifier will rank a randomly chosen positive instance higher than a randomly chosen negative one [34]. The robustness of performance was assessed via bootstrapping [35], a validation technique based on random data resampling with replacement (here, 50 times); we used the very conservative out-of-bag estimator which calculates errors on *unseen* data. To assess the entity of the difference between means of two performance distributions, a modified *t*-test was used, penalising the degrees of freedom due to sample overlap [36]. Variable importance analysis for the different machine learning techniques was carried out as follows: odds ratio/AIC with associated p-values for both multivariable and univariable logistic regression; conditional independence split rule + pruning for decision trees as implemented in *ctree* function of *party* library in R [29]; Gini index for random forest; decrease in AUC by single feature elimination for AdaBoost and support vector machines.

## Results

### Study population

Table 1 the characteristics of the study population (487 patients, 912 eyes). The percentage of males was 49.5%. The mean (std. dev.) age was 65.3 (14.9) years in males, and 70.5 (12.7) years in females. The proportion of AMD diagnoses was 22.5% in males and 31.4% in females (p < 0.0001 by a test for equality of proportions). Among AMD-diagnosed patients, dry and wet AMD had a prevalence of 9.7% and 38.1% respectively. Healthy subjects accounted for 31.6% of the study population.

**Table 1:** **Population’s characteristics**

Parameter	M	F	Total	Missing
Number of patients (%)	241 (49.5%)	246 (50.5%)	487	/
Number of eyes (%)	444 (48.7%)	468 (51.3%)	912	/
Number of healthy eyes (%)	138 (31.1%)	150 (32.1%)	288 (31.6%)	/
Age (mean+/−std)	65.3 +/− 14.9	70.5 +/− 12.7	68 +/− 14.1	/
Soft drusen positive (%)	21 (6.4%)	62 (17.9%)	83 (12.4%)	240 (26.3%)
Macular scar positive (%)	19 (5.8%)	32 (9.2%)	51 (7.6%)	237 (26%)
RPE defect/pigment mottling positive (%)	82 (25.2%)	118 (34.1%)	200 (29.8%)	240 (26.3%)
Depigmentation area positive (%)	95 (29.1%)	134 (38.7%)	229 (34.1%)	240 (26.3%)
Subretinal fluid positive (%)	79 (21.8%)	50 (13.4%)	129 (17.5%)	176 (19.3%)
Macular tickness (mean+/−std)	297.4 +/− 64.8	277 +/− 54.5	286.8 +/− 60.5	149 (16.3%)
Subretinal fibrosis positive (%)	18 (5.7%)	26 (7.5%)	44 (6.6%)	248 (27.2%)
Subretinal hemorrhage positive (%)	16 (5.2%)	19 (5.9%)	35 (5.5%)	281 (30.8%)
AMD diagnosis (%)	100 (22.5%)	147 (31.4%)	247 (27.1%)	/

Percentages of attributes are calculated considering the total of eyes with no missing values for the specific attribute in the strata (Male/Female) and total.

Table 2 shows the prevalence of non-AMD and AMD subjects, stratified by soft drusen variable, Depigmentation area and RPE defect/pigment mottling, which are the most discriminant variables for AMD. Contingency tables are available in the Additional files 1 and 2 for all the other variables.

**Table 2:** **Prevalence of diagnoses of retinal diseases in the whole population stratified by soft drusen, depigmentation area and RPE defect/pigment mottling (counting one eye as a single case)**

Disease (or healthy status)	N (%)	Soft drusen positive N (%)	Depigmentation area positive N (%)	RPE defect/pigment mottling positive N (%)
AMD	247 (27.1%)	76 (30.8%)	136 (55.1%)	125 (50.6%)
Angioid streaks	5 (0.5%)	0 (0%)	3 (60%)	1 (20%)
Central serous chorioretinopathy	69 (7.6%)	0 (0%)	21 (30.4%)	17 (24.6%)
Choroidal hemangioma	3 (0.3%)	0 (0%)	0 (0%)	0 (0%)
Diabetic retinopathy	126 (13.8%)	1 (0.8%)	18 (14.3%)	11 (8.7%)
Distrophy	24 (2.6%)	3 (12.5%)	10 (41.7%)	9 (37.5%)
Epiretinal membrane	30 (3.3%)	0 (0%)	4 (13.3%)	4 (13.3%)
Inflammatory cystoid macular edema	4 (0.4%)	0 (0%)	0 (0%)	0 (0%)
Macroaneurisma	1 (0.1%)	0 (0%)	0 (0%)	0 (0%)
Pathologic myopia	66 (7.2%)	0 (0%)	22 (33.3%)	12 (18.2%)
Retinal artery occlusion	3 (0.3%)	0 (0%)	1 (33.3%)	1 (33.3%)
Retinal vein occlusion	41 (4.5%)	0 (0%)	1 (2.4%)	2 (4.9%)
Uveitis	5 (0.5%)	0 (0%)	1 (20%)	0 (0%)

### Performance of statistical learning methods

Table 3 shows the predictive performance of models trained on the dataset (912 eyes from 487 patients) upon the bootstrap validation. Results were computed using complete cases and the three different imputation techniques described in the Methods section. Performance is shown in terms of AUC, sensitivity and specificity. In regards to AUC, random forest and logistic regression were ranked as the best, followed by AdaBoost, support vector machine, decision tree and one-rule. When considering sensitivity (the percentage of patients who are correctly identified as having AMD), support vector machine was superior, whilst random forest displayed the highest specificity (the percentage of healthy people who are correctly identified as not having AMD).

**Table 3:** **Performance of the machine learning methods in terms of average (+/− std. dev.) sensitivity, specificity and area under the receiver operating characteristic (AUC), applied to the dataset with different missing value imputation techniques (complete cases, categorical variable encoding the missingness, mean/mode imputation, and random forest imputation)**

Type of imputation on the dataset (N = 444)	Performance function	One-rule	Decision tree	Logistic regression	Random forest	AdaBoost	Support vector machine
**Complete cases**	AUC	0.74+/−0.05	0.90+/−0.03	0.93+/−0.04	**0.94+/−0.01**	0.92+/−0.02	0.92+/−0.03
	Sensitivity	0.87+/−0.10	0.88+/−0.07	0.92+/−0.03	0.90+/−0.03	0.91+/−0.02	**0.94+/−0.03**
	Specificity	0.60+/−0.18	0.74+/−0.15	0.70+/−0.08	**0.78+/−0.07**	0.71+/−0.06	0.67+/−0.07
**Categorical variable encoding the missingness**	AUC	0.73+/−0.04	0.88+/−0.02	0.91+/−0.01	**0.92+/−0.02**	0.90+/−0.01	0.89+/−0.03
	Sensitivity	0.92+/−0.07	0.88+/−0.07	0.92+/−0.03	0.91+/−0.02	0.91+/−0.03	**0.93+/−0.03**
	Specificity	0.42+/−0.05	0.61+/−0.18	0.60+/−0.07	**0.68+/−0.06**	0.60+/−0.06	0.51+/−0.07
**Mean/mode**	AUC	0.69+/−0.05	0.85+/−0.02	**0.88+/−0.02**	0.87+/−0.02	0.87+/−0.02	0.86+/−0.04
	Sensitivity	0.94+/−0.05	0.92+/−0.04	0.94+/−0.02	0.93+/−0.02	0.93+/−0.02	**0.96+/−0.02**
	Specificity	0.31+/−0.12	0.56+/−0.10	0.54+/−0.05	**0.56+/−0.05**	0.53+/−0.05	0.47+/−0.06
**Random forest**	AUC	0.79+/−0.02	0.95+/−0.02	**0.96+/−0.01**	**0.96+/−0.01**	**0.96+/−0.01**	0.94+/−0.03
	Sensitivity	**0.97+/−0.04**	0.94+/−0.04	0.96+/−0.02	0.94+/−0.02	0.95+/−0.01	0.96+/−0.01
	Specificity	0.60+/−0.06	0.78+/−0.09	0.75+/−0.05	**0.81+/−0.04**	0.76+/−0.04	0.75+/−0.05

Results are calculated on 50 bootstrap tests, using out-of-bag predictions (in bold the best performance for each characteristic).

We executed a formal *t*-test to compare shifts in the average AUCs of methods –specifically, logistic regression, support vector machine, AdaBoost and decision trees against random forest- given the current data, there was not enough evidence against the hypothesis of no difference in mean (whichever imputation method was used, all p-values were >0.3). Instead, the one-rule method had a lower mean AUC than all other methods (p < 0.0001 in all imputation scenarios).

**Figure 1:**
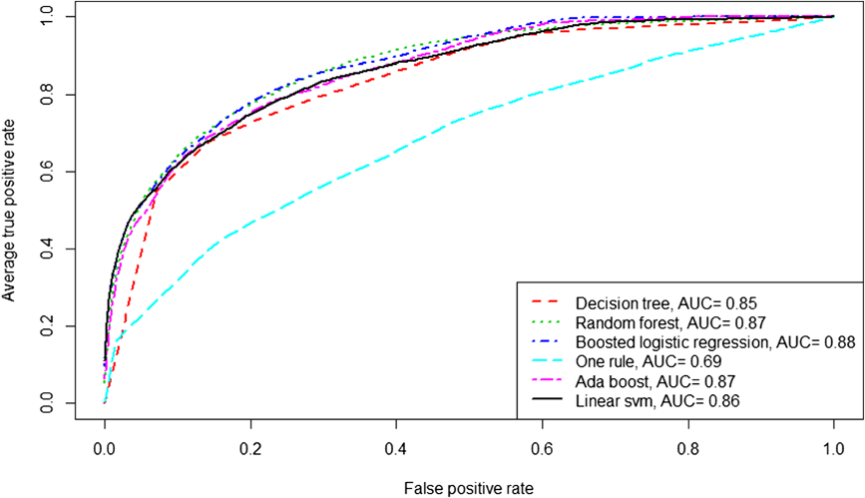
**Receiver operating characteristic curves plotting performance of different statistical learning methods, averaging results from 50 bootstrap tests (out-of-bag predictions, dataset imputing mean/mode for missing values).**

Figure 1 shows receiver operating characteristic curves for each method obtained by averaging the results from the 50 bootstrap tests. As reported in Table 3, random forest and logistic regression curves dominate the others.

Since logistic regression was not inferior to random forest in terms of AUC, we report the model fit in Table 4. Soft drusen, as expected, was the most important variable with an odds ratio (positive vs negative) of 19.3 (p < 0.0001). Other relevant variables were: subretinal fluid, subretinal hemorrage, subretinal fibrosisRPE defect/pigment mottling, depigmentation area, and age.

**Figure 2:**
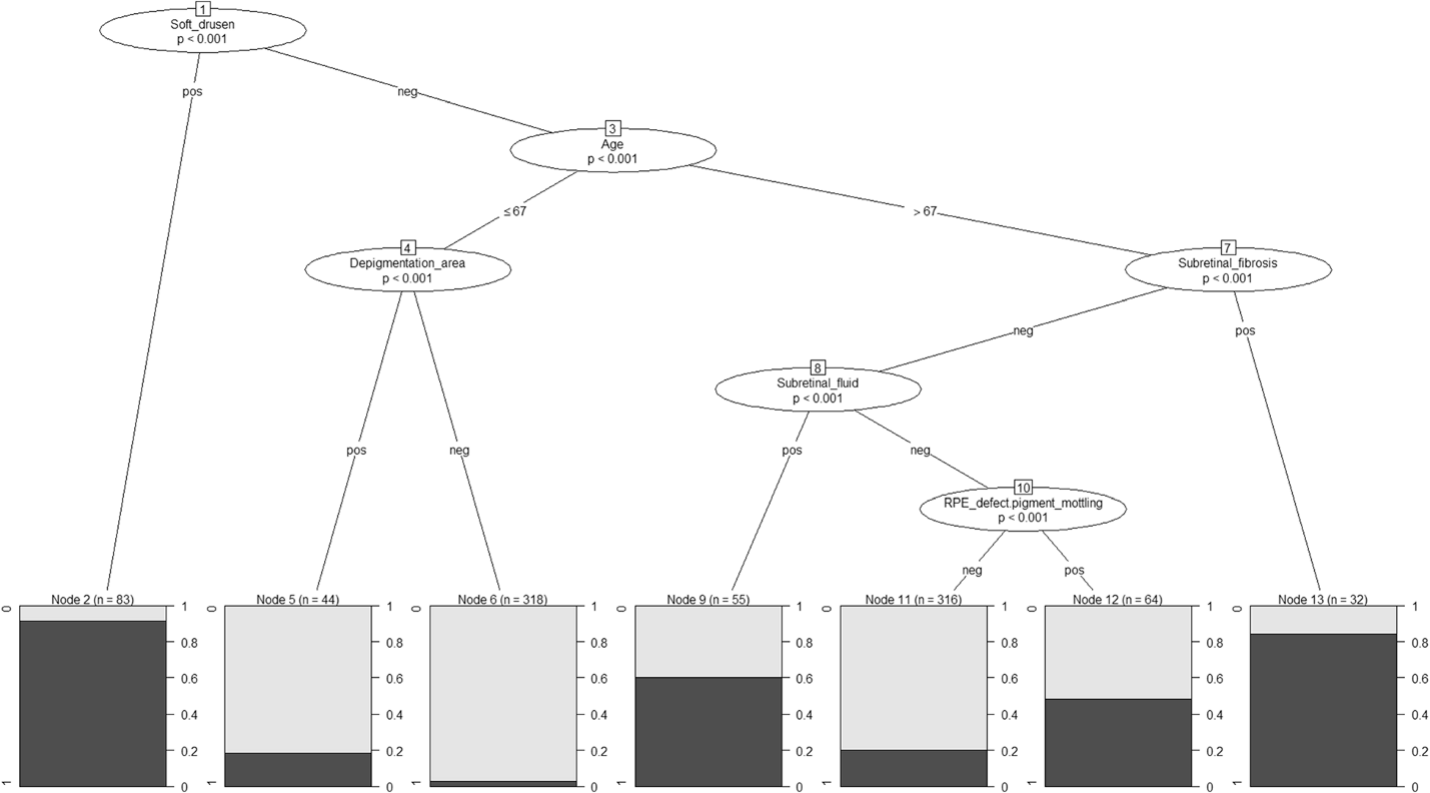
**Decision tree for the diagnosis of AMD (dataset with mean/mode imputation for missing values).** The tree is to be traversed downwards from the root node. The p-values are calculated according to a chi-square test and represent the discriminatory power of a variable in a data stratum as induced by the tree partition. Each final leaf node gives the probability of AMD diagnosis based on the prevalence in the population sub-stratum following the corresponding tree pathway induced by node splits on variable values.

As shown by the overall sensitivity, specificity and AUC results, the decision tree assures fair performance and its structure has high interpretability. The tree is shown in Figure 2 (dataset with mean/mode imputaion for missing values). The tree should be traversed from the root node downwards. Split nodes are evaluated according to the value of the variable of interest and the decision pathway to follow is the corresponding attribute value on the branch. Again, soft drusen had the highest discriminative power (76 eyes out of 83 with a positive soft drusen are diagnosed with AMD) and was selected as the root node. Following soft drusen, the other variables selected as node splits were: age, depigmentation area, subretinal fibrosis, subretinal fluid and RPE defect/pigment mottling,. Notably, the leaf nodes numbered #2, #6, 13 (corresponding to the first, third, and seventh bottom terminal nodes from the left) clearly identify sub-groups where the AMD diagnosis is straightforward (>80% with/without AMD), whilst the other leaf nodes represent sub-groups where the AMD diagnosis is present in the range of 20% to 60% (nodes #2, #9, #11, #12), thus not allowing a definitive classification.

**Table 4:** **Odds ratio from fitting the LogitBoost logistic regression on the AMD diagnosis outcome (dataset imputing missing values with population’s mean/mode)**

Variable (mode)	Odds ratio	Lower 95% CI	Upper 95% CI	P-value
Age (per year older)	1.09	1.07	1.11	**<0.0001**
Gender (M vs F)	1.05	0.71	1.57	0.7985
Soft drusen (pos vs neg)	19.30	7.82	47.65	**<0.0001**
Macular scar (pos vs neg)	1.75	0.57	5.41	0.329
RPE defect/pigment mottling (pos vs neg)	2.20	1.20	4.04	**0.0109**
Depigmentation area (pos vs neg)	1.35	0.73	2.51	0.3349
Subretinal fluid (pos vs neg)	3.21	1.70	6.08	**0.0003**
Macular tickness (per unit increase)	1.00	0.99	1.00	0.139
Subretinal fibrosis (pos vs neg)	4.60	1.39	15.24	**0.01245**
Subretinal hemorrhage (pos vs neg)	5.91	1.49	23.42	**0.01138**

Statistically significant p-values are reported in bold.

Table 5 shows variable importance ranking (mean/mode imputation) for one rule, random forest, AdaBoost and support vector machine. In agreement with logistic regression and decision tree, soft drusen and age are consistently at the top of the ranking. This is confirmed in all analyses performed with different imputation methods (see Additional files 1 and 2).

**Table 5:** **Variable importance ranking for one rule, random forest, adaboost and support vector machine (mean/mode dataset)**

Ranking	One rule	Random forest	AdaBoost	Support vector machine
1	Soft drusen	Age	Age	Age
2	Age	Soft drusen	Soft drusen	Gender
3	Depigmentation area	Macular tickness	Subretinal fluid	Soft drusen
4	RPE defect.pigment mottling	Depigmentation area	Subretinal hemorrhage	Subretinal hemorrhage
5	Subretinal hemorrhage	RPE defect.pigment mottling	RPE defect.pigment mottling	Subretinal fluid
6	Subretinal fibrosis	Subretinal fibrosis	Gender	Subretinal fibrosis
7	Macular.scar	Subretinal hemorrhage	Subretinal fibrosis	RPE defect.pigment mottling
8	Subretinal fluid	Subretinal fluid	Macular.scar	Macular.scar
9	Gender	Macular.scar	Macular tickness	Macular tickness
10	Macular tickness	Gender	Depigmentation area	Depigmentation area

## Discussion

This work investigated several machine learning approaches for deriving an automated system for AMD diagnosis, using clinical attributes identified by a medical retinal specialist during a routine visit. The study population was monitored via an Electronic Health Record employed by a single clinical practice in Genoa, Italy.

We compared “white-box” (i.e. more interpretable) vs. “black-box” (i.e. less interpretable) techniques in terms of predictive performance. We found that *higher complexity-higher performance* does not necessarily hold in all contexts and a performance-complexity compromise may be found. For example, the simplest one-rule model yielded an average of 74% AUC in experiments, whilst the more complex, fully non-linear random forest and AdaBoost yielded 92% AUC. Whilst a single variable cannot be used for reliable diagnosis, logistic regression (average AUC of 92%) and decision tree (90% average AUC) were not inferior to random forest. These two modelling techniques combined interpretability and performance, as shown through the odds ratio table and tree diagram, which can be easily followed by a clinician during the diagnostic process.

Physicians must be involved in the decision about what type of system will be used in practice because without their agreement and trust, such a system risks not to being used. Generally, from the perspective of a fully automated system, where a computer program performs all calculations, the main driver should not be the interpretability of the model, but the overall performance. For example, if a black-box ensures an increment 10% over a white-box method and if this 10% is clinically relevant (if properly validated in a prospective trial), then the choice should be obvious. But in the case where model performances are comparable, such as the results reported in this study referring, the white-box is a preferable alternative.

The methods proved to be robust to handle missing values and obtained performances did not change significantly varying the imputation methods. Although complete cases and random forest imputation yielded better performance than the other methods, we think that the most reliable analysis is the one with mean/mode imputation. In fact, the clinicians that performed the analysis suggested that the majority of missing values are likely to be clinical signs that they did not identify during encounters, and thus negative values were not recorded in the system to save time, starting from the assumption that if a sign had been identified a positive value would have been registered in the system.

This study has some limitations. The study population itself is not large (487 subjects and 912 eyes) and includes only patients from a local regional area. Although the out of bag error estimator is very conservative, a way in which the generalisation error could be challenged is by considering the study population and the diagnostic process as regionally biased: for instance, by assuming that the population of Genoa and neighbouring areas (Liguria) is different from Italy (or worldwide) and that doctors make diagnoses differently. Accordingly, it would be interesting to see how the automated diagnostic algorithms would behave on patients from other countries. This would unveil indirectly the differences in the population characteristics and in the gold-standard diagnostic procedures. Performance would be affected only by using two different systems trained on two different populations, whilst one could infer a new integrated model which takes into account such regional differences and aims at the same diagnostic ability in different settings.

A more thorough analysis of missing values could be performed in order to identify the characteristic of missingness and their relevancy. Also, further investigation on intra-patient correlation is warranted. Using two records from the same patient (i.e. both eyes) may yield to correlated observations, thus overall performance results may be affected by this correlation (higher than in reality). We carried out a series of additional experiments, not shown in this paper, using only single-patient and single-eye data (out of 912 eyes, we selected 487 eyes pertaining to 487 different patients randomly, for 10 times). The analysis on this uncorrelated data was consistent with the main results in terms of sensitivity but yielded slightly lower specificity. This is most likely due to the smaller sample size (487 vs. 912) rather than the effect of correlation in the main dataset however a larger study population is required to verify this claim. A larger population and attribute set may also help to refine the model and allow prediction of different subtypes of AMD, for instance neurovascular AMD.

## Conclusions

From a rationale point of view, the utility of the system -for now- is to determine which are the diagnostic processes followed by the physicians, since the data were cross-sectional and the diagnoses were made by doctors during visits. We found that even by using powerful nonlinear machine learning models, we could not exactly all the consistent sets of diagnostic pathways. Therefore, even in presence of standardised guidelines, physicians may follow different diagnostic routes (as those shown in the decision tree) which in some cases lead to ambiguities (see the proportions of patients with/without AMD in the tree leaves which correspond to specific variable strata). When longitudinal data and new background variables (e.g. other image processed data) will be available, an automated system will help not only in identifying such different decision paths (with an augmented information set), but also in making early diagnoses feasible and better differentiating those ambiguous subsets of patients.

In fact, we are in an era where diagnosis of AMD is most commonly pursued by image analysis, yet digital image processing techniques embedded in commercial OCT systems are still in their infancy. Imaging can be integrated with information coming from data collected during everyday clinical practice by medical retina specialists. From a technological perspective, implementing such a diagnostic model into a computer program would not be a hurdle, and using a multi-platform language (e.g. Java or Python) could facilitate the integration into Electronic Health Record systems coming from different vendors. If the model were also one of those white-box (logistic regression or decision tree in this case), a graphical user interface showing the diagnostic pathway or variable importance could be provided. Such a program could be used in real-time by physicians as a support to diagnosis as well as for educational purposes.

## Electronic supplementary material

Additional file 1: **Cross tabulation between AMD and all covariates.** (TXT )

Additional file 2: **Variable importance derived for each missing value imputation technique.** (XLSX )
